# PB.4. Ultrasound false negative preoperative axillary assessment in breast cancer patients undergoing sentinel node biopsy

**DOI:** 10.1186/bcr3734

**Published:** 2014-11-03

**Authors:** L Grosvenor, M Al-Attar, D Lister, G McDonald, S Kaneri, N Hartley, M Hoosein, L Sundaram, E Denton

**Affiliations:** 1 Breast Care Centre, Glenfield Hospital, UHL, Leicester, UK

## Introduction

Concern was raised by our local pathology unit regarding an increase in the number of positive sentinel node biopsies (SNB). As an imaging group we did not have the evidence base to refute the concerns raised and therefore undertook a 6-month retrospective review of unit performance.

## Methods

Pathology provided a database list of all patients they regarded as positive SNB (false negative US assessment). The period covered was 6 months (1 November 2012 to 30 April 2013). This was referenced against all axillary surgeries during the same period.

## Results

The pathology database extended to 98 patients. Multiple duplications were identified and correctly identified preoperative C5 axillae were excluded. A final cohort of 43 positive SNB (false negative US) assessments were identified from the 266 SNB procedures performed in the time period. The 13 operators included locums, trainees, multi-professionals and external consultant staff. All operators had at least one false negative assessment of the axilla. Positive nodal histology was reported for 14 cases as micro-metastases or isolated tumour cells (ITC) and in 29 cases as macro-metastases/extranodal spread or multiple involved nodes. Eleven per cent of SNBs were considered false negative US assessments (29/266). Our NPV calculates at 83.8% (43 patients = 223/266) for all surgical positive axillae and 89% with exclusion of micro-metastases/ITC.

## Conclusion

No individual performance or unit concern was outside the published literature ranges. Individual clinical cases of concern were identified for peer review/clinical governance. Imaging review has led to consolidation of the working practice and an ongoing prospective audit with continual review. However, the balance between adequate assessment, capacity and resource needs defining. How good is good enough?

